# In vitro and in vivo functions of T cells produced in complemented thymi of chimeric mice generated by blastocyst complementation

**DOI:** 10.1038/s41598-022-07159-7

**Published:** 2022-02-25

**Authors:** Kazuto Yamazaki, Kenji Kubara, Satoko Ishii, Peng Li, Ryo Dairiki, Taro Hihara, Yuta Ishizuka, Yukina Izumi, Minoru Kumai, Tsutomu Kamisako, Hiroyoshi Ishizaki, Hideyuki Sato, Hideki Masaki, Naoaki Mizuno, Kaoru Mitsuhashi, Masashi Ito, Sanae Hamanaka, Tomoyuki Yamaguchi, Motoo Watanabe, Fumihiro Sugiyama, Hiromitsu Nakauchi

**Affiliations:** 1 Tsukuba Research Laboratories, Eisai Co., Ltd., 5-1-3, Tokodai, Tsukuba, Ibaraki 300-2635 Japan; 2 KAN Research Institute, Inc., 6-8-2 Minatojima-minamimachi, Chuo-ku, Kobe, Hyogo 650-0047 Japan; 3 Division of Stem Cell Therapy, Institute of Medical Science, The University of Tokyo, 4-6-1 Shirokanedai, Minato-ku, Tokyo, 108-8639 Japan; 4 Laboratory Animal Resource Center, Transborder Medical Research Center, Faculty of Medicine, University of Tsukuba, 1-1-1, Tennodai, Tsukuba, Ibaraki 305-8575 Japan; 5 Department of Genetics, Institute for Stem Cell Biology and Regenerative Medicine, Stanford University School of Medicine, 265 Campus Drive, Stanford, CA 94305 USA

**Keywords:** Developmental biology, Stem cells

## Abstract

Blastocyst complementation is an intriguing way of generating humanized animals for organ preparation in regenerative medicine and establishing novel models for drug development. Confirming that complemented organs and cells work normally in chimeric animals is critical to demonstrating the feasibility of blastocyst complementation. Here, we generated thymus-complemented chimeric mice, assessed the efficacy of anti-PD-L1 antibody in tumor-bearing chimeric mice, and then investigated T-cell function. Thymus-complemented chimeric mice were generated by injecting C57BL/6 (B6) embryonic stem cells into *Foxn1*^*nu*/*nu*^ morulae or blastocysts. Flow cytometry data showed that the chimeric mouse thymic epithelial cells (TECs) were derived from the B6 cells. T cells appeared outside the thymi. Single-cell RNA-sequencing analysis revealed that the TEC gene-expression profile was comparable to that in B6 mice. Splenic T cells of chimeric mice responded very well to anti-CD3 stimulation in vitro; CD4^+^ and CD8^+^ T cells proliferated and produced IFNγ, IL-2, and granzyme B, as in B6 mice. Anti-PD-L1 antibody treatment inhibited MC38 tumor growth in chimeric mice. Moreover, in the chimeras, anti-PD-L1 antibody restored T-cell activation by significantly decreasing PD-1 expression on T cells and increasing IFNγ-producing T cells in the draining lymph nodes and tumors. T cells produced by complemented thymi thus functioned normally in vitro and in vivo. To successfully generate humanized animals by blastocyst complementation, both verification of the function and gene expression profiling of complemented organs/cells in interspecific chimeras will be important in the near future.

## Introduction

Blastocyst complementation is a promising method of using pluripotent stem cells (PSCs) to generate humanized animals for the purpose of establishing novel in vivo models for drug development and the production of organs for transplantation. In this methodology, PSCs are injected into blastocysts that harbor spontaneous or genetically modified mutations causing the loss of target organs or cells. Injected donor PSCs take over the defective host’s niche made vacant by the mutation, and they develop into normal organs or cells instead of those with mutation-related deficits. The following organs and cells have thus far been generated successfully through blastocyst complementation: T cells and B cells^1^, lens^2^, hematopoietic cells^3^, pancreas^4–7^, kidney^6–9^, thymus^6,10,11^, endothelial cells and hematopoietic cells^7,12^, lung^13^, forebrain (neocortex and hippocampus)^14^, and liver^7,15^.

For the successful application of blastocyst complementation to in vivo animal models for drug development and regenerative medicine, the complemented organs must have not only normal anatomical and cytohistological architecture but also normal physiological functions. In pancreas-complemented chimeras (PSCs → *Pdx1*^−/−^ blastocysts), glucose tolerance tests and transplantation of complemented islets into diabetic animals have indicated a functional response of the complemented pancreata to blood glucose^4,5^. Mori et al.^13^ produced chimeric mice in which the lungs were complemented (*Shh*^Cre/+^ *Fgfr2*^flox/flox^), and both the lungs and trachea were complemented (*Shh*^Cre/+^ *Ctnnb1*^flox/flox^) by conditional blastocyst complementation. They demonstrated normal lung function in these chimeric mice, including normal resistance, compliance, and elastance of the respiratory system and resistance of the conducting airway in response to methacholine. Chang et al.^14^ produced forebrain-complemented mice by using *Emx1*–Cre;*Rosa26*–DTA (diphtheria toxin A) blastocysts as hosts. Novel-object-recognition and Morris water maze testing indicated that the chimeras’ learning and memory were intact.

The thymus is a specialized primary lymphoid organ responsible for the differentiation of progenitor T cells (thymocytes) into mature T cells expressing self-MHC (major histocompatibility complex)-restricted and self-tolerant T-cell receptors (TCRs). Thymic epithelial cells (TECs) play a very important role in T-cell repertoire selection—that is, positive and negative selection. Forked-box N1 (FOXN1) is a transcriptional factor that is a master regulator of the differentiation of TECs^16,17^, and *Foxn1* mutations result in athymic animals, such as *Foxn1*^*nu*/*nu*^ mice^18^ and *Foxn1*^*rnu*/*rnu*^ rats^19^. Therefore, animals that are thymus-complemented by using *Foxn1* mutant blastocysts would be useful in building interesting animal models in immunology and oncology. As mentioned above, thymus-complemented animals have been reported by several groups^6,10,11^; complementation of thymi by donor cells and the presence of T cells have been reported. However, there have been few detailed analyses of T-cell function. Moreover, to our knowledge, gene expression analysis of complemented organs at the single-cell level compared with wild-type normal organs has not been reported.

Here, we generated thymus-complemented chimeric mice by complementation using *Foxn1*^*nu*/*nu*^ blastocysts. For the first time, we investigated the gene expression of complemented thymi at a single-cell level by conducting single-cell RNA-sequencing (scRNA-seq), focusing particularly on TECs. Furthermore, we examined the functions of T cells of thymus-complemented chimeric mice, not only in vitro, but also in vivo pharmacologically, by utilizing a tumor transplantation model to evaluate the effects of an immune check inhibitor for their future application to cancer immunology.

## Results

### Generation of thymus-complemented mouse chimeras (B6 ESC^CAG-EGFP^ → ***Foxn1***^***nu***/***nu***^ and B6 ESC^CAG-AG^ → ***Foxn1***^***nu***/***nu***^)

**Figure 1:**
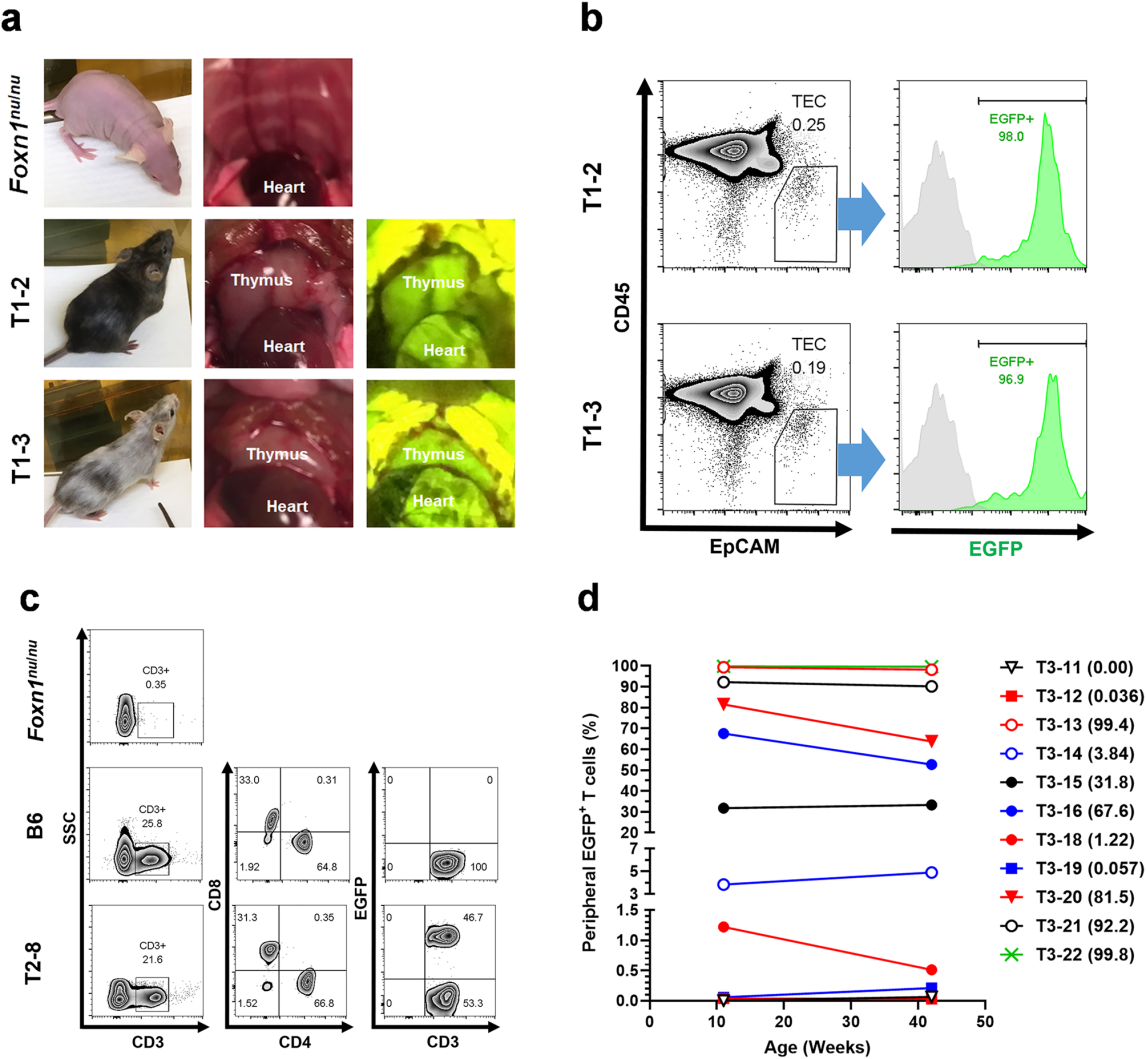
General characterization of thymus-complemented chimeric mice that were generated by blastocyst complementation—that is, injection of C57BL/6 (B6) embryonic stem cells expressing CAG-EGFP (ES^CAG-EGFP^) into nude KSN/Slc (*Foxn1*^*nu*/*nu*^) blastocysts. (**a**) Appearances and thymi of a nude (*Foxn1*^*nu*/*nu*^) mouse and two B6 ESC^CAG-EGFP^ → *Foxn1*^*nu*/*nu*^ chimeric mice (mice nos. T1-2 and T1-3). The nude mouse was athymic and both chimeras had EGFP-positive thymi. (**b**) Flow cytometry analysis of thymic cells derived from the two chimeric mice. Thymic cells were sorted against CD45 and EpCAM intensity, and the fractions of thymic epithelial cells (TECs; CD45^−^EpCAM^+^) were extracted (percentages of TECs were 0.25% and 0.19%, respectively), and then sorted against EGFP fluorescence. Almost all TECs of the chimeras were EGFP positive (98.0% and 96.9% in nos. T1-2 and T1-3, respectively). B6 control, gray; chimeras, green. (**c**) Generation of peripheral T cells in B6 ESC^CAG-EGFP^ → *Foxn1*^*nu*/*nu*^ chimeras. Representative flow cytometry analysis of peripheral T cells of *Foxn1*^*nu*/*nu*^, B6, and chimeric (mouse no. T2-8) mice, demonstrated as zebra plots. CD3^+^ cells are marked in boxes, with their percentages in the left column. CD8^+^ or CD4^+^ cells and EGFP^+^CD3^+^ or EGFP^−^CD3^+^ cells, respectively, of the B6 mouse and the chimera are indicated in the middle and right columns. Percentage of cells in each of four divided sections is shown. In the case of chimera no. T2-8, about 50% of the T cells were EGFP^+^. (**d**) Changes in percentages of peripheral EGFP^+^ T cells of 11 chimeras between 11 and 42 weeks of age with varying chimerism. The identification numbers of the individual chimeric mice are indicated at right, with percentages of EGFP^+^ peripheral T cells at 11 weeks of age in parentheses. There were no significant differences in chimerism between the ages (*P* = 0.1541 by paired two-tailed Student’s *t*-test).

Chimeric mice were generated by injection of enhanced green fluorescent protein (EGFP)- and Azami green (AG)-positive C57BL/6 (B6) embryonic stem cells (ESCs) into KSN/Slc and CD1-*Foxn1*^*nu*/*nu*^ blastocysts, respectively. The mice varied in nude–hairy or black–white coat color chimerism (i.e., in the contribution of the donor) in appearance. At autopsy, chimeric mice had thymi regardless of the degree of chimerism. It seemed, however, that chimeric mice with lower chimerism in appearance had smaller thymi (n = 8 for B6 ESC^CAG-EGFP^ → *Foxn1*^*nu*/*nu*^; n = 6 for B6 ESC^CAG-AG^ → *Foxn1*^*nu*/*nu*^). Representative examples are shown in Suppl. Fig. 1. Figure 1a indicates the outward appearance and complemented EGFP^+^ thymi of two B6 ESC^CAG-EGFP^ → *Foxn1*^*nu*/*nu*^ chimeric mice with different coat color chimerism (mice nos. T1-2 and T1-3), compared with those of a nude (*Foxn1*^*nu*/*nu*^) mouse. Flow cytometry (FCM) analysis of TECs (CD45^−^EpCAM^+^) showed that almost all TECs of the chimeric mice were derived from EGFP^+^ donors (Fig. 1b). In the periphery, we observed T cells (CD3^+^) with various types of chimerism in the chimeric mice. The higher chimerism in appearance (larger hairy and black coat-color area), the higher percentages of donor-derived (EGFP^+^ or AG^+^) peripheral T cells (Suppl. Fig. 2). In addition, they possessed CD8 single-positive and CD4 single-positive T cells, as in B6 mice, whereas there were almost no T cells in the nude mice (Fig. 1c). Peripheral T cell numbers of 11 B6 ESC^CAG-EGFP^ → *Foxn1*^*nu*/*nu*^ chimeric mice were measured to examine relationships between chimerism and T cell numbers. Among 11 chimeras, one chimera showed extremely high number of T cells (~ 8500 /µL) (Suppl. Fig. 3a). Suppl. Fig. 3b depicts a regression line between percentages of EGFP^+^ T cells and peripheral T cell numbers, after excluding the high value judged by Grubbs's test for outliers (*P* < 0.05). There was no positive or negative correlation between them. Peripheral T cell numbers of chimeras were not significantly different from those of B6 mice (Suppl. Fig. 3c). Age-dependent changes in chimerism of peripheral T cells were examined in 11 chimeric mice between 11 and 42 weeks of age. There were no significant differences in T-cell chimerism between the ages (Fig. 1d).

### scRNA-seq analysis of thymic cells of B6 and B6 ESC^CAG-EGFP^ → *Foxn1*^*nu*/*nu*^ chimeric mice

**Figure 2:**
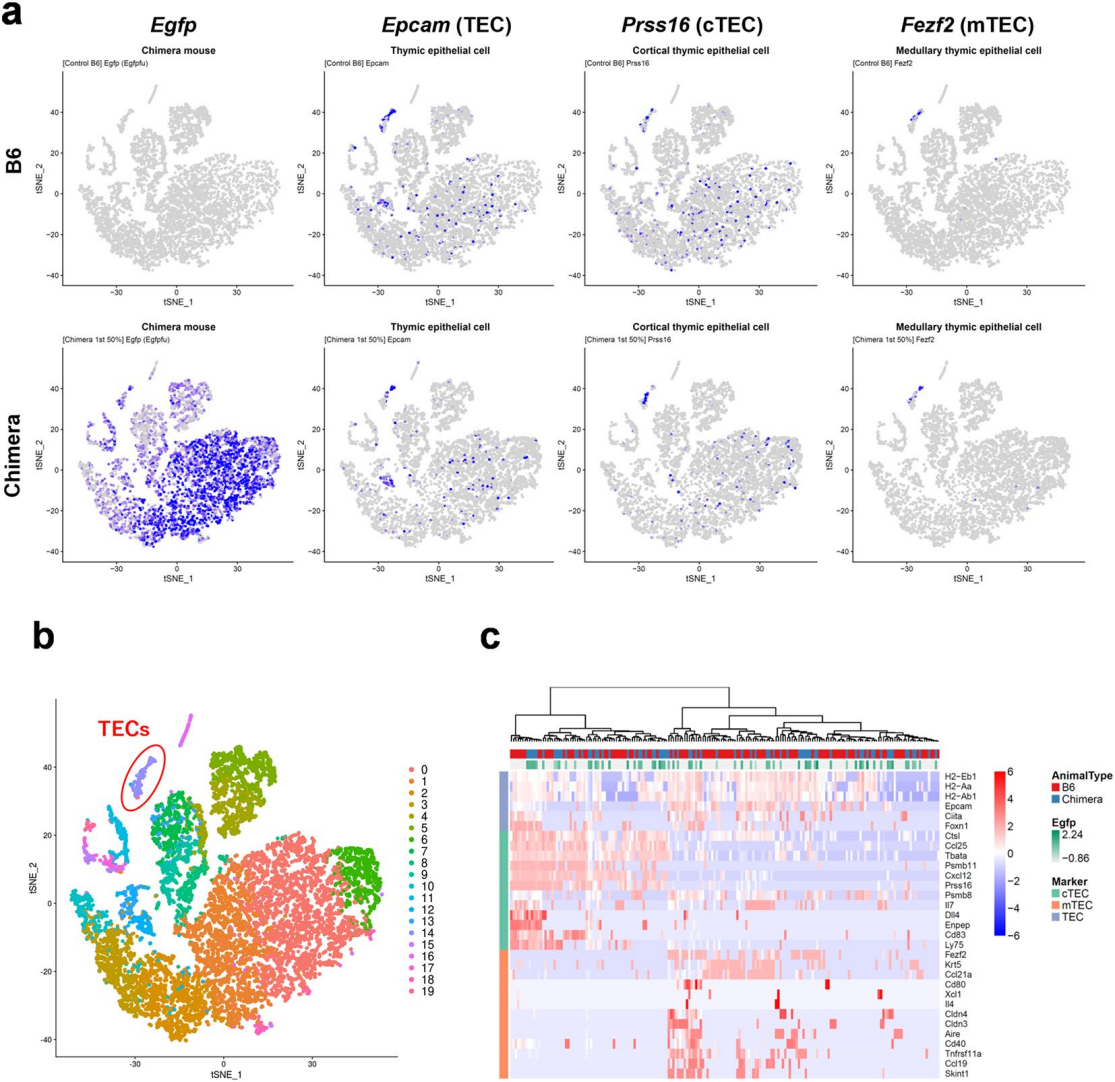
Single-cell transcriptomic profiling of thymus-complemented chimeric mice. (**a**) Projection of whole thymic cells derived from C57BL/6NCrSlc (B6) and B6 ESC^CAG-EGFP^ → KSN/Slc (*Foxn1*^*nu*/*nu*^) chimeric mice onto two principal dimensions by using t-distributed stochastic neighbor embedding. *Egfp*, *Epcam*, *Prss16*, and *Fezf2* expression was plotted on cells derived from B6 (upper row) and chimeric mice (lower row). *Epcam*, *Prss16*, and *Fezf2* are markers of thymic epithelial cells (TECs), cortical TECs (cTEC), and medullary TECs (mTEC), respectively. (**b**) Classification of clustering of cells, resulting in a total of 20 clusters. From the distribution of expression of TEC markers such as *Epcam*, as indicated in (**a**), a cluster of TECs numbered 14 was detected (red oval). (**c**) Heatmap of single cells extracted from TEC cluster 14 in B6 and thymus-complemented mice. The degree of *Egfp* expression is shown as green shading in the plot. Cells were categorized by the common TEC, cTEC, and mTEC markers in the left column. Gene expression levels are indicated as scaled normalized unique molecular identified counts.

To compare the gene expression patterns of complemented thymi of chimeric mice with those of normal mice, we conducted scRNA-seq analysis of whole thymi. After drawing a t-distributed stochastic neighbor embedding (t-SNE) chart, we plotted TEC marker gene expression, namely the expression of epithelial cell adhesion molecule (*Epcam*) for TECs, thymus-specific serine protease (TSSP; *Prss16*) for cortical TECs (cTECs), and FEZ family zinc finger 2 (*Fezf2*) for medullary TECs (mTECs), as well as *Egfp* expression (Fig. 2a). The three marker genes had specific distributions. Classification based on gene expression similarity resulted in a total of 20 clusters (Fig. 2b), among which cluster 14 was identified to be enriched with TEC gene markers, including interleukin 7 (*Il7*), thymoproteasome subunit β5t (*Psmb11*), and autoimmune regulator (*Aire*), in addition to the abovementioned three marker genes. We therefore regarded cluster 14 as the TEC cluster.

We extracted cells from the TEC cluster to create a heatmap of TEC-related gene expression (Fig. 2c). In the heatmap, the genes were classified into three categories on the basis of the patterns of common TEC, cTEC, and mTEC gene marker expression, along with *Egfp* expression. We observed correspondence of the origins of TECs (chimera or B6) with *Egfp* expression (presence or absence), reflecting the FCM results in Fig. 1b that almost all TECs in the chimeras were EGFP positive. In addition, this analysis revealed heterogeneity of TECs from adult chimeric and B6 mice, as reported in mouse embryos (E12.5–E18.5) and newborns (postnatal day 0)^20^. TECs from B6 and chimeric mice were uniformly distributed in the heatmap, indicating that the two types of mice had similar heterogeneity of TECs.

### In vitro T-cell-proliferation assays using splenic T cells of B6 ESC^CAG-EGFP^ → *Foxn1*^*nu*/*nu*^ chimeric mice

**Figure 3:**
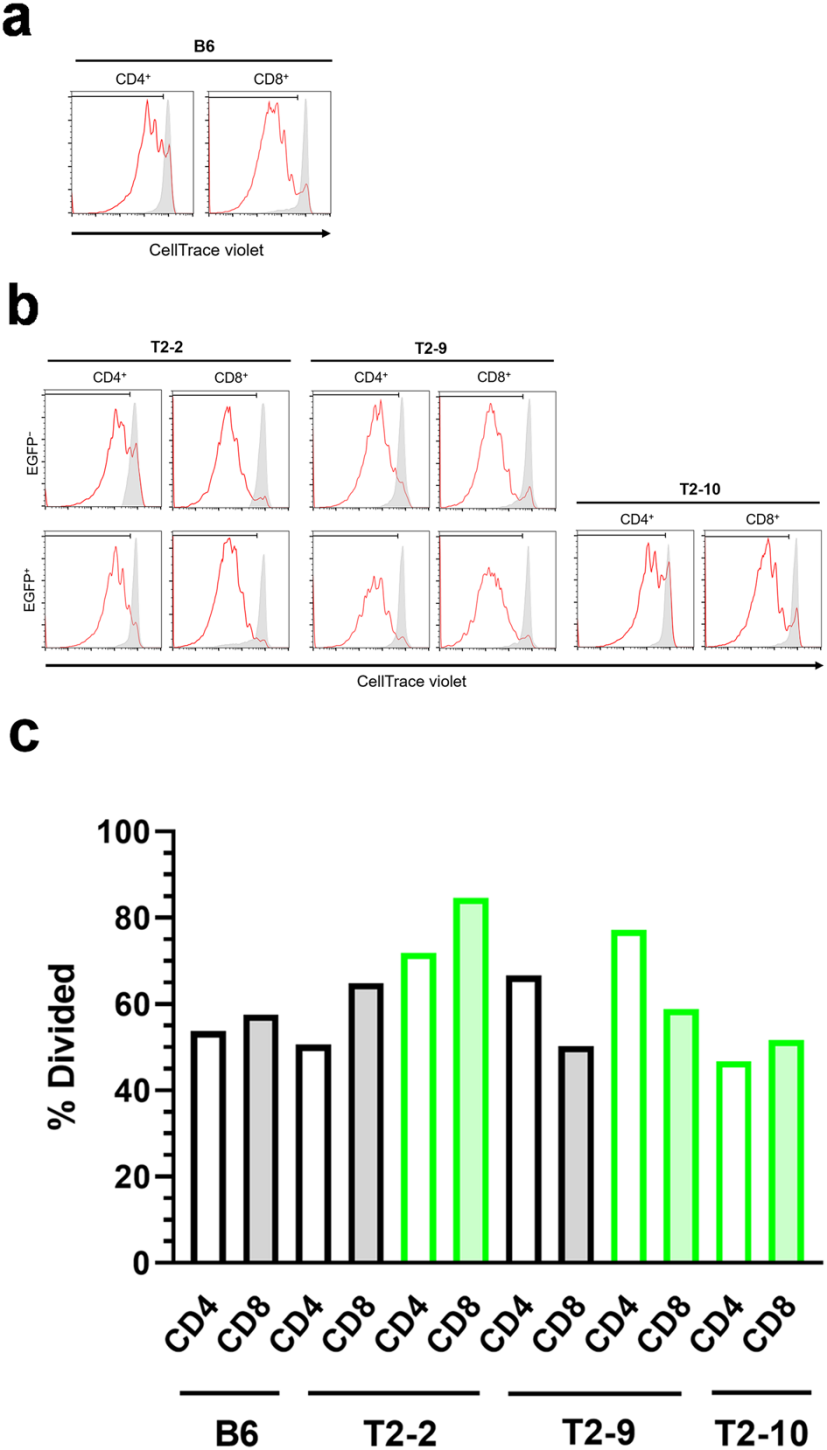
Anti-CD3/CD28 antibody-induced proliferation of splenic T cells of C57BL/6NCrSlc (B6) and B6 ESC^CAG-EGFP^ → KSN/Slc (*Foxn1*^*nu*/*nu*^) chimeric mice. Splenic T cells were labeled with CellTrace Violet, activated with anti-CD3/CD28 antibody beads for 3 days, and then underwent flow cytometry. (**a**) Histograms of CellTrace Violet staining in CD4^+^ or CD8^+^ T cells derived from a B6 mouse. Stimulated, red lines; unstimulated, gray shading. (**b**) Histograms of CellTrace Violet staining in EGFP^−^CD4^+^ and EGFP^+^CD4^+^ T cells and EGFP^−^CD8^+^ and EGFP^+^CD8^+^ T cells derived from B6 ESC^CAG-EGFP^ → *Foxn1*^*nu*/*nu*^ chimeric mice (nos. T2-2, T2-9, and T2-10). Stimulated, red lines; unstimulated, gray shading. (**c**) Percentages of divided EGFP^−^CD4^+^ and EGFP^+^CD4^+^ T cells and EGFP^−^CD8^+^ and EGFP^+^CD8^+^ T cells derived from chimeric mice, as well as CD4^+^ and CD8^+^ T cells of B6 mice, as determined by using CellTrace Violet staining and FlowJo software. Black borders on bars indicate EGFP^−^ T cells and green borders indicate EGFP^+^ T cells. Open boxes indicate CD4^+^ T cells and color-shaded (gray or green) boxes indicate CD8^+^ T cells.

For functional analysis of T cells produced by complemented thymi, splenic T cells isolated from three chimeric mice with different chimerism were stimulated with anti-CD3/CD28 antibody beads to evaluate the proliferation potential of CD4^+^ and CD8^+^ T cells by priming through costimulatory signals. Figure 3a is a representative histogram showing the proliferation of CD4^+^ and CD8^+^ T cells of B6 mice. We plotted the proliferation patterns of CD4^+^ and CD8^+^ T cells, categorized into EGFP^−^ and EGFP^+^, in three chimeras (mice nos. T2-2, T2-9, and T2-10) (Fig. 3b) and in B6 mice. Both donor- and host-derived splenic T cells of the chimeras proliferated. The chimerism of splenic T cells from chimeras nos. T2-2, T2-9, and T2-10 after 72 h of stimulation was 87.3%, 10.1%, and 98.8%, respectively—almost the same values of those of splenic T cells before the stimulation (see “Methods” section). Splenic T cells of chimeric mice proliferated upon stimulation with the antibodies in a manner similar to those isolated from B6 mice, regardless of the origin of the T cells (host or donor) (Fig. 3c).

### In vitro T-cell-activation assays using splenic T cells of B6 ESC^CAG-AG^ → *Foxn1*^*nu*/*nu*^ chimeric mice

**Figure 4:**
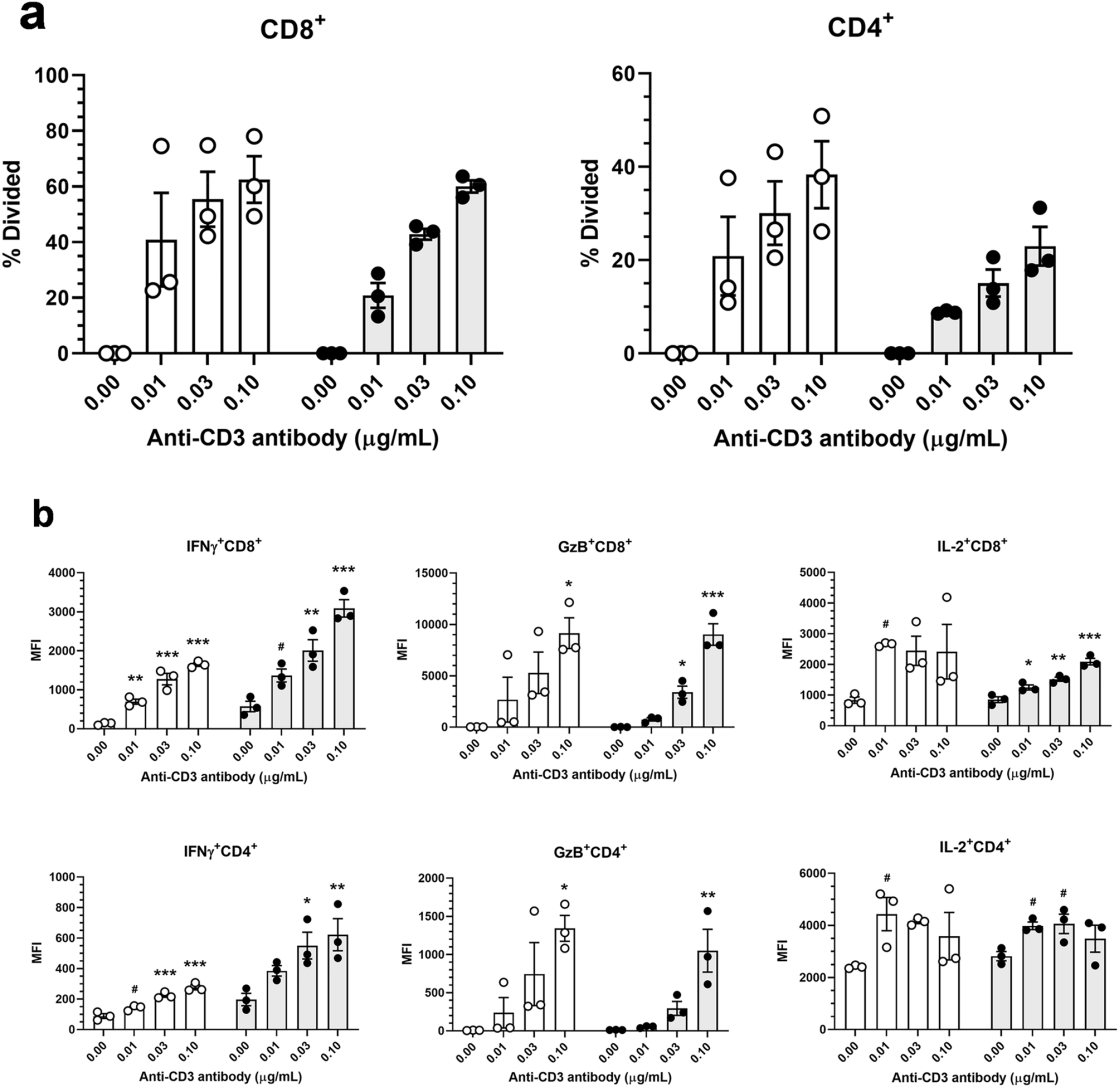
Cytokine and granzyme B (GzB) production in T cells derived from C57BL/6 J (B6) and B6 ESC^CAG-Azami-green^ → CD1-*Foxn1*^*nu*/*nu*^ chimeric mice. Splenic T cells were labeled with CellTrace Violet and activated with anti-CD3 antibody (0, 0.01, 0.03, or 0.1 μg/mL) for 3 days, and then underwent flow cytometry. (**a**) Percentages of divided cells, as determined by using CellTrace Violet staining and FlowJo software. Open circles, B6 mice; filled circles, chimeric mice. Mean ± S.E.M. n = 3 per group. (**b**) Mean fluorescence intensity (MFI) of interferon-γ (IFNγ), GzB, and interleukin 2 (IL-2) positivity of CD4^+^ and CD8^+^ T cells. Values were analyzed by Dunnett’s multiple comparisons test among the B6 groups (open circles) or chimera groups (filled circles). *, **, and *** indicate *P* < 0.05, *P* < 0.01, and *P* < 0.001, respectively, and ^#^ indicates *P* < 0.10, vs. the vehicle group. Mean ± S.E.M. n = 3 per group.

Next, to elucidate further the in vitro function of splenic T cells of chimeric mice, we used FCM analysis to investigate the production of cytokines (interferon-γ (IFNγ) and interleukin-2 (IL-2)) and a cytotoxic protease (granzyme B (GzB)) after stimulation with anti-CD3 antibody. In both B6 and chimeric mice, antibody-concentration-dependent proliferation of CD4^+^ and CD8^+^ T cells was observed (Fig. 4a). The mean fluorescence intensity (MFI) of IFNγ^+^CD4^+^, GzB^+^CD4^+^, IFNγ^+^CD8^+^, or GzB^+^CD8^+^ T cells was significantly increased in an anti-CD3-antibody-concentration-dependent manner. IL-2^+^CD8^+^ T cells likewise showed a concentration-dependent increase, whereas the MFI of IL-2^+^CD4^+^ T cells peaked at 0.01 or 0.03 μg/mL, showing a bell-shaped change (Fig. 4b).

### Tumor transplantation experiments using B6 ESC^CAG-AG^ → *Foxn1*^*nu*/*nu*^ chimeric mice

**Figure 5:**
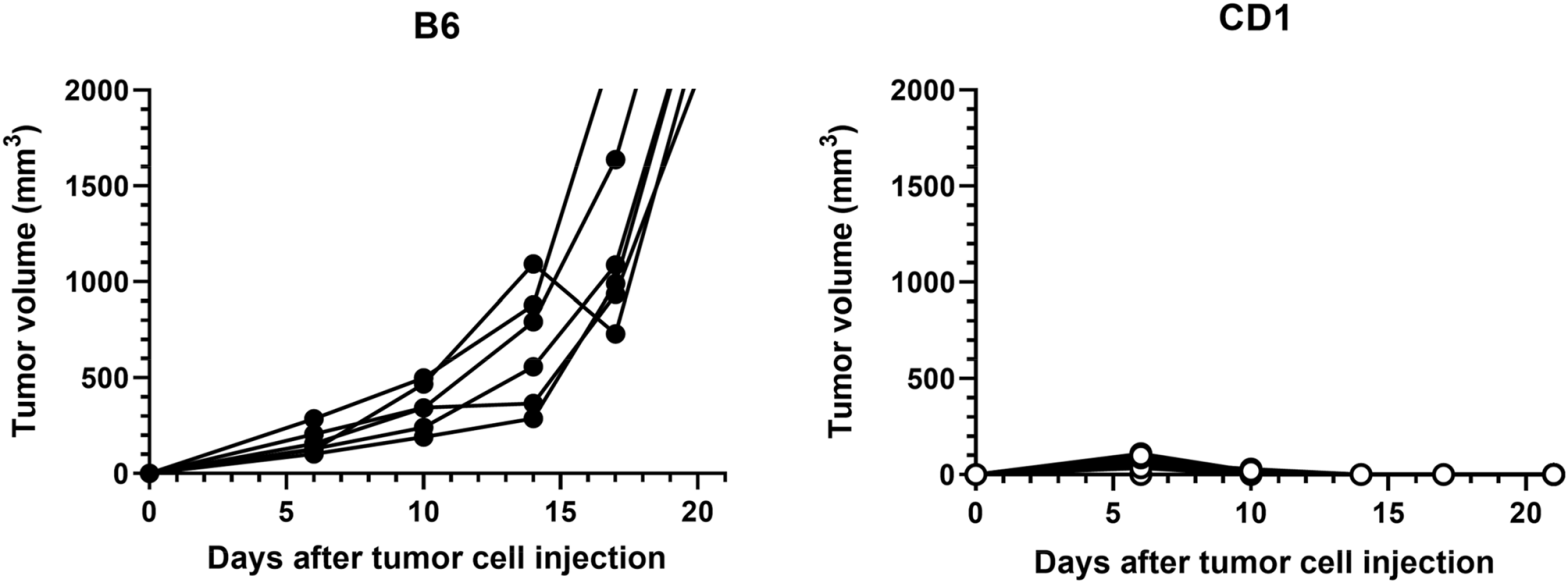
Tumor cell inoculation into C57BL/6NCrl (B6) and CD1 mice. B6-derived cancer cells (MC38 cells) were transplanted into the right flank at 1 × 10^6^ cells/head subcutaneously (day 0), and tumor volumes were measured on days 6, 10, 14, 17, and 21. Tumor growth curves of six B6 (filled circles) and 10 CD1 (open circles) mice. Tumor cells were engrafted and grew rapidly, exceeding 2000 mm^3^ in all B6 mice, whereas all CD1 mice rejected the cells.

**Figure 6:**
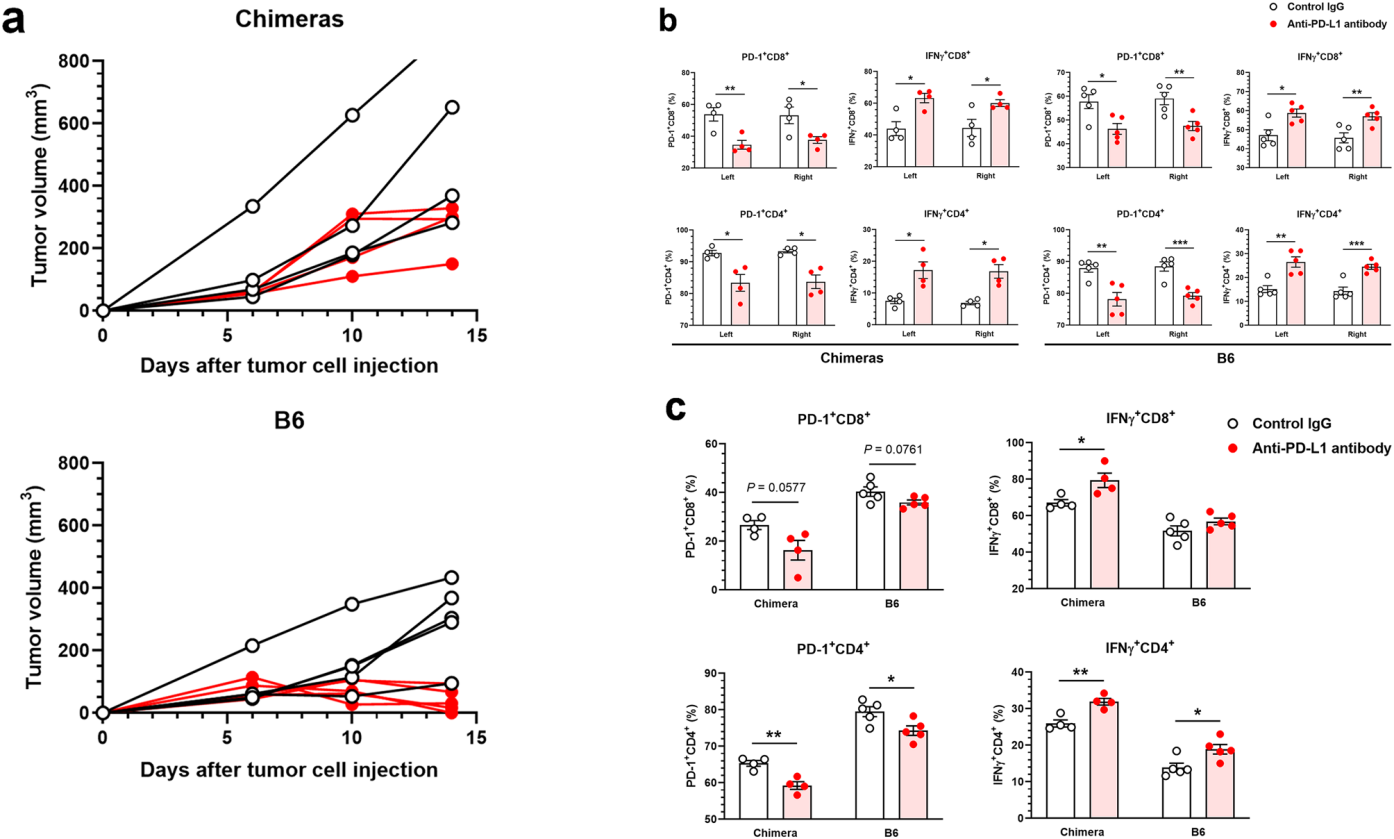
Antitumor effects of anti-PD-L1 antibody on tumors engrafted in C57BL/6 J (B6) and B6 ESC^CAG-Azami-green^ → CD1-*Foxn1*^*nu*/*nu*^ chimeric mice. MC38 cells (B6-derived cancer cells syngenic to the donor of the thymus-complemented chimeric mice), were inoculated subcutaneously into the right flank of B6 mice and chimeras at 1 × 10^6^ cells/head (day 0), and tumor volumes were measured on days 6, 10, and 14. Anti-PD-L1 antibody (50 μg/100 μL/mouse) or control IgG (50 μg/100 μL/mouse) was administered intraperitoneally on days 7 and 10. On day 14, draining lymph nodes (dLNs) and tumors were harvested for flow cytometry (FCM) analysis. (**a**) Tumor growth curves of individual chimeric mice and B6 mice (open black circles, control IgG; filled red circles, anti-PD-L1 antibody). (**b**) Percentages of PD-1^+^CD4^+^ and PD-1^+^CD8^+^ T cells and IFNγ^+^CD4^+^ and IFNγ^+^CD8^+^ T cells in the left and right dLNs of chimeras and B6 mice. (**c**) FCM analysis of tumor-infiltrating lymphocytes (TILs) of chimeras and B6 mice. Percentages of PD-1^+^CD4^+^ and PD-1^+^CD8^+^ TILs and IFNγ^+^CD4^+^ and IFNγ^+^CD8^+^ TILs were compared between the control and anti-PD-L1 antibody treatments. Values were analyzed by *F*-test, followed by an unpaired two-tailed Student’s *t*-test or two-tailed Welch’s *t*-test between treatments. *, **, and *** indicate *P* < 0.05, *P* < 0.01, and *P* < 0.001, respectively. Means ± S.E.M. n = 4 for each group of chimeras; n = 5 for each group of B6 mice.

First, we monitored the growth of inoculated MC38 cells in B6 and CD1 mice for 21 days. In B6 mice, the tumors grew rapidly and their volumes in all transplanted mice exceeded 2000 mm^3^ by day 21 (Fig. 5). In contrast, all CD1 mice completely rejected the MC38 cells within 14 days (Fig. 5). As we had confirmed the normal functions of T cells produced by complemented thymi in vitro, we next investigated their functions in vivo. For this purpose, we evaluated the antitumor effects of anti-PD-L1 antibody in an MC38-transplantation model. B6 and chimeric mice were injected with MC38 cells (day 0) and then treated with anti-PD-L1 antibody or control IgG (days 7 and 10). Notably, anti-PD-L1 antibody administration seemed to suppress tumor growth in both B6 mice and the chimeras (Fig. 6a). Furthermore, we used FCM to investigate T-cell activation in the draining lymph nodes (dLNs), as well as T-cell-mediated target-cell-killing capacity in tumor-infiltrating lymphocytes (TILs), on day 14 after cancer cell injection. Anti-PD-L1 antibody treatment significantly enhanced T-cell activation by downregulating programmed cell-death 1 (PD-1) expression and upregulating IFNγ production by CD4^+^ and CD8^+^ T cells in the dLNs of both B6 and chimeric mice (Fig. 6b). In addition, in CD4^+^ and CD8^+^ TILs, we observed that IFNγ production was increased and PD-1 expression was decreased by anti-PD-L1 antibody in the chimeras (Fig. 6c). The similar effects of anti-PD-L1 antibody were also detected in B6 mice. In general, anti-PD-L1 antibody had similar effects in terms of both T-cell activation in dLNs and T-cell-killing capacity in TILs in chimeras and B6 mice. Taken together, these findings show that we successfully generated thymus-complemented chimeric mice. We demonstrated that thymus-complemented chimeric mice and normal B6 mice had similar genetic profiles and similar thymic functions, as well as similar thymus-derived peripheral T-cell responses. Blastocyst complementation technology will be invaluable in generating humanized animals for both research and commercial applications in regenerative medicine and drug development.

## Discussion

The thymus is a primary lymphoid organ for the differentiation of thymocytes into mature T cells and maintenance of the pool of functional T cells. TECs are essential in the process of maturation of T cells. cTECs and mTECs play different roles, in positive and negative selection, respectively, in the T-cell maturation process. FOXN1 is a transcriptional factor that is a master regulator of TEC differentiation in the fetal period and of TEC homeostasis in the postnatal stage^16,17^. The gene responsible in nude mice (*nu*/*nu*)^18^ and nude rats (*rnu*/*rnu*)^19^ is *Foxn1*. In nude mouse fetuses, the thymus primordium forms normally and thymic epithelial precursor cells are present. Afterwards, however, the nude thymic anlage never develops to support T cells, and it remains as nonfunctional cystic thymic rudiments. As a result, nude mice are athymic and lack normal T-cell populations^21^.

In terms of the physiological roles of the thymus, thymus complementation by blastocyst complementation can be expected to generate an in vivo unique model for drug development, particularly in the field of cancer immunology. Determining whether or not T cells work functionally in chimeras is important for the application of such a model. Although intra- and interspecific thymus complementation has been reported^6,10,11^, there have been few detailed reports on the functions of T cells generated by complemented thymi. Therefore, we examined the in vitro and in vivo functions of T cells of B6 ESC → *Foxn1*^*nu*/*nu*^ chimeric mice. In addition, we conducted scRNA-seq analysis of complemented TECs, which control the development and maturation of T cells, to investigate whether their gene expression profile was comparable to that in TECs of B6 mice.

We generated thymus-complemented mouse chimeras in which peripheral T cells with varying chimerism were produced by blastocyst complementation. Previously, Müller et al.^10^ indicated that mTECs and cTECs in mESCs → *Foxn1*^*nu*/*nu*^ chimeric mice were constituted from the progeny of engrafted mESCs by RT-PCR. However, the characteristics of complemented mTECs and cTECs are not proved yet. In this study, scRNA-seq analysis of total thymic cells showed similar t-SNE clustering patterns in B6 and chimeric mice, suggesting that there was normal development of complemented thymi, including T cells, at the gene expression level. The heatmap for cells in the TEC clusters of normal and chimeric mice demonstrated that the TECs of the chimera were derived from donor B6 ESCs. Therefore, we demonstrate that complemented TECs not only express TECs’ marker genes, but also show heterogeneity comparable to wild-type mice. TECs express several genes that are prerequisites for T-cell selection and maturation. For example, *Psmb11* and *Prss16* are expressed for the positive selection of CD8^+^^22^ and CD4^+^ T cells^23^, respectively, in cTECs. *Aire* is expressed for the negative selection of T cells in mTECs^24^. We confirmed that these TEC representative genes were expressed in similar ways in B6 and chimeric mice. TEC gene expression of the chimera, comparable to that in B6, could thus indicate normal differentiation of thymocytes and T cells in complemented thymi. As mentioned above, TECs control T-cell development from immature thymocytes to mature T cells. Conversely, thymocytes control TEC expansion and differentiation. These bidirectional interactions between thymocytes and TECs are referred to as thymic crosstalk^25,26^. For example, RANKL (receptor activator of nuclear factor kappa-Β ligand; tumor necrosis factor ligand superfamily member 11, *Tnfsf11*) provided by positively selected CD4^+^ thymocytes regulates mTEC differentiation via RANK (*Tnfrsf11a*) expressed on mTECs^27,28^. Therefore, the normal gene expression profiles of TECs of the chimera suggest that there is normal development of thymocytes and T cells.

To investigate the functions of T cells generated by complemented thymi in chimeras, we first assessed splenic T-cell proliferation in chimeras after stimulation with anti-CD3/CD28 antibodies, in comparison with that in B6-derived T cells. We observed comparable proliferation in chimera- and B6-derived T cells, as well as in donor (EGFP^+^ *Foxn1*^+/+^)- and host-derived T cells (EGFP^−^ *Foxn1*^*nu*/*nu*^), suggesting that *Foxn1* genotypes do not affect the function of T cells. There was also comparable production of IFNγ, IL-2, and GzB by splenic T cells stimulated with anti-CD3 antibody in chimeras and B6 mice. Notably, anti-CD3 antibody induced T-cell-receptor-mediated proliferation and activation in a concentration-dependent manner. Collectively, these findings indicated that the development of thymus-derived T lymphocytes works normally in thymus-complemented chimeric mice created by blastocyst complementation.

The host immune system is one of the most important elements for protection from tumor development and control of tumor growth. Tumor growth is controlled mainly by T lymphocytes. CD8^+^ cells are regarded as being a uniform population of cells that largely and quickly secret IFNγ and a protease, GzB, which can kill tumor cells. In addition, antigen-specific CD4^+^ T cells showing a Th1 IFNγ-producing phenotype were detected against tumor growth; this was identified recently as an additional mechanism for controlling tumor growth^29^. T cells become activated through both antigen-receptor signaling and CD28 costimulatory signaling. During activation, these T cells express PD-1, which interacts with its ligand PD-L1, resulting in the inhibition of T-cell proliferation and activation. In the tumor microenvironment (TME), PD-1 is expressed mainly on activated T cells, whereas PD-L1 is expressed on several types of tumor cells and antigen-presenting cells. Under these circumstances, tumor cells escape from host immunity by utilizing the immune checkpoint system. Preclinical and clinical studies have demonstrated the efficacy of immune checkpoint inhibitors (that is, anti-PD-1 antibody and anti-PD-L1 antibody) against several cancers. Currently, several antibodies have been marketed (e.g., nivolumab and pembrolizumab against PD-1; atezolizumab and avelumab against PD-L1), and antibodies against the PD-1 pathway are revolutionizing cancer immunotherapy^30,31^.

To assess the tumor-inhibitory effect of T cells in thymus-complemented chimeric mice, we evaluated the pharmacological efficacy of anti-PD-L1 antibody on B6-derived tumors, syngeneic to the donor, implanted into B6 ESC → *Foxn1*^*nu*/*nu*^ chimeras, in consideration of the future application of thymus-complemented models to cancer immunology. Anti-PD-L1 antibody administration suppressed tumor growth in the chimeras and resulted in the increased abundance of IFNγ^+^CD8^+^ and IFNγ^+^CD4^+^ T cells and decreased abundance of PD-1^+^CD8^+^ and PD-1^+^CD4^+^ T cells in dLNs. The same phenomena were also observed in TILs. We postulate that the immune checkpoint interaction between PD-1 on T cells generated by complemented thymi and PD-L1 on tumor cells worked normally. In addition, the activation of CD4^+^ T cells by anti-PD-L1 antibody in the chimeras suggested that TSSP (PRSS16) in complemented TECs functions normally, because TSSP is crucial for the antitumoral role of CD4^+^ T cells^32^. This speculation was supported by our finding of normal expression of *Prss16* in cTECs of the chimera by the scRNA-seq analysis described above.

Here, we generated thymus-complemented chimeric mice by blastocyst complementation—that is, by injection of B6 ESCs into *Foxn1*^*nu*/*nu*^ blastocysts—and characterized the mice’s T cells in vitro and in vivo*.* We also performed gene expression profiling of their complemented TECs. We showed that T cells produced by complemented thymi worked normally, as they did in B6 mice. We speculate that the normal function of the chimeras’ T cells was reflected by the normal gene expression patterns of the TECs, which were the targets of complementation in the chimeras. It was reported that T cell lymphopenia and reduced proliferative responses of T cells to mitogens in patients with thymic hypoplasia, such as DiGeorge syndrome^33,34^. As we observed that a tendency that chimeras with lower chimerism had smaller thymi, it is likely that the chimerism has an effect on T cell numbers and functions. Our results showed, however, that the chimerism did not affect peripheral T cell numbers. The chimerism of the chimeric mice used as a tumor-engrafted model was extremely low, but T cells of the chimeras functioned normally comparable to those of B6 mice in vivo. Taken together with these findings, it is shown that the chimerism of thymus-complemented mice affects the thymus size, but not T cell numbers or functions.

For oncogenesis research and anticancer drug development, humanized mice (e.g., human hematopoietic stem cell (HSC)-engrafted immunodeficient mice) have been utilized and bettered (e.g., transgenic for human leukocyte antigen (HLA)). However, there are some limitations of the models, such as occurrence of graft-versus-host disease after HSC engraftment, difficulty of construction of the TME^35^. In the principle of blastocyst complementation, it has the potentials to generate novel types of humanized mouse models which can break the current limitations. Thus, we could generate a novel model in which cells involved in the TME (e.g., endothelial cells, macrophages, and adipocytes^36^) are also complemented, in addition to T cells and TECs, by using hosts harboring *Foxn1*^*nu*/*nu*^ and (an)other mutant gene(s). For example, it is presumed that we may produce chimeric mice which have not only donor-derived vascular endothelial cells and myeloids, but also donor-derived T cells selected by donor-derived TECs, by using double *Foxn1*^*nu*/*nu*^ and *Flk-1*^−/−^ mutant hosts^12^. The results of this study are fundamental for the future application of blastocyst complementation to cancer immunology.

Blastocyst complementation technology will advance the generation of humanized animals for research and commercial applications in regenerative medicine and drug development. To achieve this, validation of the function and gene expression patterns, which are influenced by xenogeneic barriers (e.g., ligand–receptor incompatibility, cell adhesion incompatibility), of complemented organs and cells of interspecific chimeras through comparison with normal donor animals or humans will be important in the future.

## Methods

### Mice

KSN/Slc (*Foxn1*^*nu*/*nu*^) and C57BL/6NCrSlc mice were obtained from Japan SLC (Hamamatsu, Shizuoka, Japan). C57BL/6NCrl, C57BL/6J, Crlj:CD1-*Foxn1*^*nu*^ (CD1-*Foxn1*^*nu*/*nu*^), and Crl:CD1 (CD1) mice were purchased from Charles River Laboratories Japan (Yokohama, Kanagawa, Japan). MCH/ICR mice were purchased from CLEA Japan Inc. (Tokyo, Japan). The C57BL/6 substrains used in this study are abbreviated as B6 in the text. Animal care and experimental procedures were performed in animal facilities accredited by the Health Science Center for Accreditation of Laboratory Animal Care and Use of the Japan Health Sciences Foundation. All protocols were approved by the Institutional Animal Care and Use Committee of Eisai Co., Ltd. (Tokyo, Japan) and KAN Research Institute, Inc. (Kobe, Hyogo, Japan), and of the Institute of Medical Science, University of Tokyo (Tokyo, Japan). All methods were performed in accordance with relevant guidelines and regulations, and the study complied with ARRIVE guidelines (Animal Research: Reporting of In Vivo Experiments).

### Generation of mouse ESC lines, and cell culture

A B6 ESC^CAG-EGFP^ line was derived from blastocysts obtained from C57BL/6N female mice mated with C57BL/6N-Tg male mice (CAG-EGFP) (Japan SLC) and maintained as previously described^5^.

*Rosa26*–CAG–fNeo–H2B–AG–dNeo, a Cre-reporter mouse ESC line, was generated by gene targeting. A reporter gene cassette containing the following components was constructed in a Bluescript SK + (Stratagene, Santa Clara, CA, USA) backbone: CAG promoter–LoxP–Neo–polyA (as a stop cassette)–LoxP–histone H2B (H2B)–AG–polyA. The *Rosa26* 5ʹ and 3ʹ arms (1.7 kb and 6 kb, respectively) were amplified with the following primers and then assembled with the reporter gene cassette to construct the targeting vector: 5′ arm: 5ʹ-GAG GAA TTC CCG GGA GGC CCA ACG CGG CGC CAC GGC GTT TC-3ʹ/5ʹ-GAG CTC GAG AAG ACT GGA GTT GCA GAT CAC GAG GG-3ʹ; 3ʹ arm: 5ʹ-GAG GTC GAC AGA TGG GCG GGA GTC TTC TGG GCA GG-3ʹ/5ʹ-GAG GGT ACC CCC TGA CAA AAG GGA TGC CCA ATT CC-3ʹ. A knock-in allele was generated by homologous recombination in the B6J-S1^UTR^ ESC line^37^ in accordance with standard procedures. To generate the *Rosa26*–CAG–H2B–AG line constitutively expressing H2B–AG (ESC^CAG-AG^), a Cre expression vector was transfected into *Rosa26*–CAG–fNeo–H2B–AG ESCs to delete the LoxP-flanked Neo cassette. The ESC^CAG-AG^ cell line was cultured in accordance with reference^37^.

### Generation of chimeric mice by blastocyst complementation

To generate B6 ESC^CAG-EGFP^ → *Foxn1*^*nu*/*nu*^ chimeric mice, three to five B6 ESC^CAG-EGFP^ cells were injected into each KSN/Slc morula embryo. These embryos were cultured in N2B27 medium for 24 h up to the blastocyst stage and then transferred into the uteri of pseudopregnant ICR female mice (2.5 days post-coitum).

Female CD1-*Foxn1*^*nu*/*nu*^ mice were superovulated by intraperitoneal injection of HyperOva (0.1 mL; Kyudo, Tosu, Saga, Japan), followed by intraperitoneal injection of 7.5 IU human chorionic gonadotrophin (hCG) 48 h later. After being injected with the hCG, the female mice were mated with male CD1-*Foxn1*^*nu*/*nu*^ mice. *Foxn1*^*nu*/*nu*^ mouse embryos were collected in M2 medium (Sigma-Aldrich, St. Louis, MO, USA) at the two-cell stage. They were then transferred into KSOM medium (ARK Resource*,* Kumamoto, Japan) and cultured for 48 h. Chimeric embryos were generated by microinjection of B6 ESC^CAG-AG^ cells into *Foxn1*^*nu*/*nu*^ blastocysts. An XYClone laser system (Hamilton Thorne, Beverly, MA, USA) was used to open the zona pellucida before the injection of ESCs into blastocysts. The injected embryos were transferred into the uteri of pseudopregnant recipient MCH/ICR mice.

### scRNA-seq of thymi of B6 and B6 ESC^CAG-EGFP^ → *Foxn1*^*nu*/*nu*^ chimeric mice

Thymi were harvested from chimeric and C57BL/6NCrSlc mice and then digested with 0.2 mg/mL Liberase TM (Thermolysin Medium) (Sigma-Aldrich) and 0.2 mg/mL DNase I (Sigma-Aldrich) at 37 °C for 30 min. Red blood cells were removed with BD Pharm Lyse Lysing Buffer (BD Biosciences, San Jose, CA, USA). scRNA-seq libraries of thymic cells were prepared by using a Chromium Controller (10× Genomics, Pleasanton, CA, USA) and a Chromium Single Cell 3ʹ GEM, Library & Gel Bead Kit v3 (10× Genomics) in accordance with the manufacturer’s instructions. Sequencing was performed with an Illumina HiSeq 150PE configuration (Illumina, San Diego, CA, USA) in accordance with the manufacturer’s instructions.

### Preparation of transcript count data

The single-cell RNA-seq data derived from FASTQ files were processed with CellRanger software (10× Genomics, version 3.1.0) in accordance with the manufacturer’s instructions. Mouse genome data (refdata-cellranger-mm10-3.0.0.tar.gz, corresponding to GRCm38.93 in NCBI; https://support.10xgenomics.com/single-cell-gene-expression/software/release-notes/build#mm10_3.0.0) and *EGFP* sequence data (http://radiation-japan.info/pdf/green_mouse) were used as the reference genome to generate matrix files containing cell barcodes and transcript counts.

### Normalization and visualization of transcript count data

Normalization and visualization of the transcript count data were performed by using R (version 3.6.0; R Foundation for Statistical Computing, Vienna, Austria) and the Seurat package (https://satijalab.org/seurat/, version 3.1.4). The transcript data were normalized to obtain nUMI (normalized unique molecular identified) counts and were scaled by using the NormalizeData and ScaleData functions of the Seurat package. Highly variable genes and significant principal components across the normalized count data were identified and extracted for subsequent analysis by using either the FindVariableFeatures or the RunPCA function of the Seurat package. To illustrate the similarities between cells, dimensionality reduction was performed on the data with the t-SNE algorithm implemented in the RunTSNE function of the Seurat package. The single-cell data were plotted in two-dimensional space on the basis of the reduced matrix generated by the t-SNE algorithm and the expression levels of tissue-specific marker genes.

### Identification of thymus epithelial cluster and visualization of marker expression

Cells were classified into 20 clusters according to the similarity of their expression patterns by using the FindNeighbors and FindClusters functions of the Seurat package. We mapped the expression of TEC gene markers (*Epcam*, *Prss16*, and *Fezf2*) into the t-SNE chart and identified a cluster enriched with TECs. The expression data of the cells included in the thymus epithelial cluster were extracted, and the gene expression values of either TEC markers (*Ciita*, *Epcam*, *Foxn1*, *H2-Aa*, *H2-Ab1*, and *H2-Eb1*), or cTEC markers (*Ccl25*, *Cd83*, *Ctsl*, *Cxcl2*, *Dll4*, *Enpep*, *Il7*, *Ly75*, *Prss16*, *Psmb11*, *Psmb8*, and *Tbata*), or mTEC markers (*Aire*, *Ccl19*, *Ccl21a*, *Cd40*, *Cd80*, *Cldn3*, *Cldn4*, *Fezf2*, *Il4*, *Krt5*, *Skint1*, *Tnfrsf11a*, and *Xcl1*), as well as of *Egfp*, were chosen for visualization by heatmap. Gene expression levels were indicated in scaled nUMI counts, which were standardized to a mean of 0 and standard deviation of 1 in individual genes. Hierarchical clustering was performed by using the hclust function implemented in the fastcluster module (https://cran.r-project.org/web/packages/fastcluster/, version 1.1.25) with Ward’s linkage algorithm and the Manhattan distance metric. The heatmap figure was illustrated by using the heatmap function in the NMF (Non-negative Matrix Factorization) package (https://cran.r-project.org/web/packages/NMF/, version 0.22.0).

### In vitro proliferation of splenic T cells of B6 ESC^CAG-EGFP^ → *Foxn1*^*nu*/*nu*^ chimeric mice stimulated with anti-CD3/CD28 antibodies

Three B6 ESC^CAG-EGFP^ → *Foxn1*^*nu*/*nu*^ chimeric mice were used (mice nos. T2-2, T2-9, and T2-10). The mice were euthanized by cervical dislocation and their spleens were collected. After the cells had been filtered through a 70-μm-mesh cell strainer they were treated in BD Pharm Lyse Lysing Buffer to remove red blood cells. At this point, the chimerism of splenic T cells (percentage of EGFP^+^CD3^+^ cells) was 85.0%, 15.8%, and 99.9% for T2-2, T2-9, and T2-10, respectively. Next, T cells were sorted with a Mouse Pan T Cell Isolation Kit (Miltenyi Biotec, Bergisch Gladbach, Germany). T cells were then labeled with CellTrace violet (ThermoFisher Scientific, Waltham, MA, USA) and 1 × 10^5^ cells were treated with 1 × 10^5^ Dynabeads Mouse T-Activator CD3/CD28 (Thermo Fisher Scientific) at 37 °C for 72 h. After the incubation, the cells underwent FCM analysis. Dead and live cells were sorted by using propidium iodide (Dojindo, Kumamoto, Japan). Percentages of divided cells were calculated as the number of cells that went into division/the number of cells at the start of culture × 100% by using FlowJo software version 10.7.2 (Tree Star, Ashland, OR, USA).

### In vitro cytokine and granzyme production by splenic T cells of B6 ESC^CAG-AG^ → *Foxn1*^*nu*/*nu*^ chimeric mice stimulated with anti-CD3 antibody

Three B6 and three B6 ESC^CAG-AG^ → *Foxn1*^*nu*/*nu*^ chimeric mice were used (mice nos. K1-22, K1-23, and K1-28). The chimerism of their peripheral T cells (AG^+^CD3^+^ cells) was 0.17%, 0.47%, and 0.74% for K1-22, K1-23, and K1-28, respectively. The mice were euthanized by cervical dislocation and their spleens were collected. After the cells had been filtered through a 70-µm-mesh cell strainer they were centrifuged at 500*g* for 5 min and then resuspended in 2 mL of BD Pharm Lyse Lysing Buffer to remove red blood cells. Total splenic cells were labeled with CellTrace violet (Thermo Fisher Scientific), and then 1 × 10^6^ cells were treated with 0.01, 0.03, or 0.1 µg/mL anti-mouse CD3 antibody (clone 145-2C11; BioLegend, San Diego, CA, USA) at 37 °C for 48 h. After the incubation, the cells underwent FCM analysis.

### Tumor cell line

The B6 colon adenocarcinoma cell line MC38 was obtained from Kerafast (Boston, MI, USA). MC38 cells were cultured in DMEM (FUJIFILM Wako, Osaka, Japan) containing 10% FBS (Gibco, Grand Island, NY, USA), 2 mM glutamine (Gibco), 0.1 mM non-essential amino acids (Gibco), 1 mM sodium pyruvate (Gibco), 10 mM HEPES (Gibco), and 1× penicillin–streptomycin (FUJIFILM Wako) at 37 °C under a 5% CO_2_ atmosphere.

### Transplantation of B6-derived cancer cells into B6 and CD1 mice

Ten CD1 and six C57BL/6NCrl mice were injected subcutaneously into the right flank with 1 × 10^6^ MC38 cells in 100 μL of HBSS (Gibco). Tumor sizes were measured 6, 10, 14, and 21 days after the injection; their volumes were calculated as length × width^2^ × 0.5 (mm^3^). When the tumor volume was > 2000 mm^3^, the mice were euthanized by cervical translocation.

### Anti-tumor effects of anti-PD-L1 (CD274) antibody in a B6-derived tumor model in B6 ESC^CAG-AG^ → *Foxn1*^*nu*/*nu*^ chimeric mice

We examined whether anti-PD-L1 antibody had anti-tumor effects in a syngenic tumor model in chimeric mice similar to B6 mice, basically in accordance with the study by Gong et al.^38^. Ten C57BL/6J mice and eight B6 ESC^CAG-AG^ → *Foxn1*^*nu*/*nu*^ chimeric mice were inoculated with MC38 cells as mentioned above (day 0). They were then injected with control IgG (50 μg/100 μL/head; GoInVivo Purified Rat IgG2b, κ, clone RTK4530, catalog no. 400666, BioLegend) (n = 5 for B6; n = 4 for chimeras) or anti-PD-L1 antibody (50 μg/100 μL/head; GoInVivo Purified anti-mouse CD274, Rat IgG2b, κ, clone 10F.9G2, catalog no. 124328, BioLegend) (n = 5 for B6; n = 4 for chimeras) intraperitoneally on day 7 and 10. The chimerism (the percentage of peripheral AG^+^ T cells [CD3^+^]) of the individual chimeric mice in each group was 0%, 0%, 0.015%, and 0.10% in the control IgG group and 0%, 0%, 0%, and 0.039% in the anti-PD-L1 antibody group. This indicated that we had mainly estimated the in vivo function of host (CD1)-derived T cells that had been selected by donor (B6)-derived TECs. On day 14, mice were killed by cervical translocation and the dLNs and tumors were collected for FCM analysis.

### FCM

To analyze peripheral cells, blood was collected from the caudal vein, mixed with saline (Otsuka Pharmaceutical, Tokyo, Japan) containing heparin (10 units/mL; AY Pharmaceuticals, Tokyo, Japan), and treated with BD Pharm Lyse Lysing Buffer to remove erythrocytes. Thymi were minced and digested at 37 °C for 30 min with RPMI 1640 (FUJIFILM Wako) containing 0.2 mg/mL Liberase TM (Sigma-Aldrich) and 0.2 mg/mL DNase I (Roche). After filtration through a 70-µm-mesh cell strainer, cells were incubated in 1 mL of Pharm Lyse Lysing Buffer to remove red blood cells.

For FCM of TILs, tumor tissues were minced into 2- to 3-mm pieces and digested by using a Tumor Dissociation Kit (mouse) (Miltenyi Biotec) and a gentleMACS Octo Dissociator (Miltenyi Biotec). After the removal of red blood cells by using BD Pharm Lyse Lysing Buffer, TILs were isolated with CD45 (TIL) MicroBeads (mouse) (Miltenyi Biotec) and a MiniMACS Separator (Miltenyi Biotech). Isolated 1.5 × 10^6^ TILs were treated with 13 ng/mL phorbol 12-myristate 13-acetate (Sigma-Aldrich), 333 ng/mL ionomycin (Sigma-Aldrich), and BD GolgiStop (BD Biosciences) at 37 °C for 4 h. After this stimulation, TILs were incubated with Mouse BD Fc Block (BD Biosciences) for 10 min, followed by staining with antibodies against cell-surface markers. Next, TILs were fixed with 4% paraformaldehyde phosphate buffer solution (FUJIFILM Wako), permeabilized by using BD Perm/Wash Buffer (BD Biosciences), and then stained with antibodies against intracellular cytokines. In addition, cells were isolated from dLNs for FCM. They were blocked and stained as described above. Stained cells were analyzed by using an SH800 cell sorter (Sony, Tokyo, Japan), a BD LSRFortessa Flow Cytometer (BD Biosciences), or a BD FACSymphony Flow Cytometer (BD Biosciences) with FlowJo software. Peripheral T cell numbers in blood were calculated by using CountBright Absolute Counting Beads for flow cytometry (ThermoFisher Scientific) according to its protocol. Antibodies used are listed in Supplementary Table 1.

### Statistical analysis

Graph drawing and statistical analysis were performed by using GraphPad Prism version 9.0.2 (GraphPad Software, San Diego, CA).

## Supplementary Information

Supplementary Information.

## Data Availability

The scRNA-seq data presented in this study have been deposited in DRA (DDBJ Sequence Read Archive; https://www.ddbj.nig.ac.jp/dra/index.html) and are accessible through accession number DRA013040 (https://ddbj.nig.ac.jp/public/ddbj_database/dra/fastq/DRA013/DRA013040/).
