# Supplementary material for: In vitro and in vivo functions of T cells produced in complemented thymi of chimeric mice generated by blastocyst complementation
Source: Sci Rep. 2022 Feb 25;12:3242. doi: 10.1038/s41598-022-07159-7 (PMC8881621; doi:10.1038/s41598-022-07159-7)
Supplement: Supplementary file 1 — Supplementary Information. [file 41598_2022_7159_MOESM1_ESM.pdf]

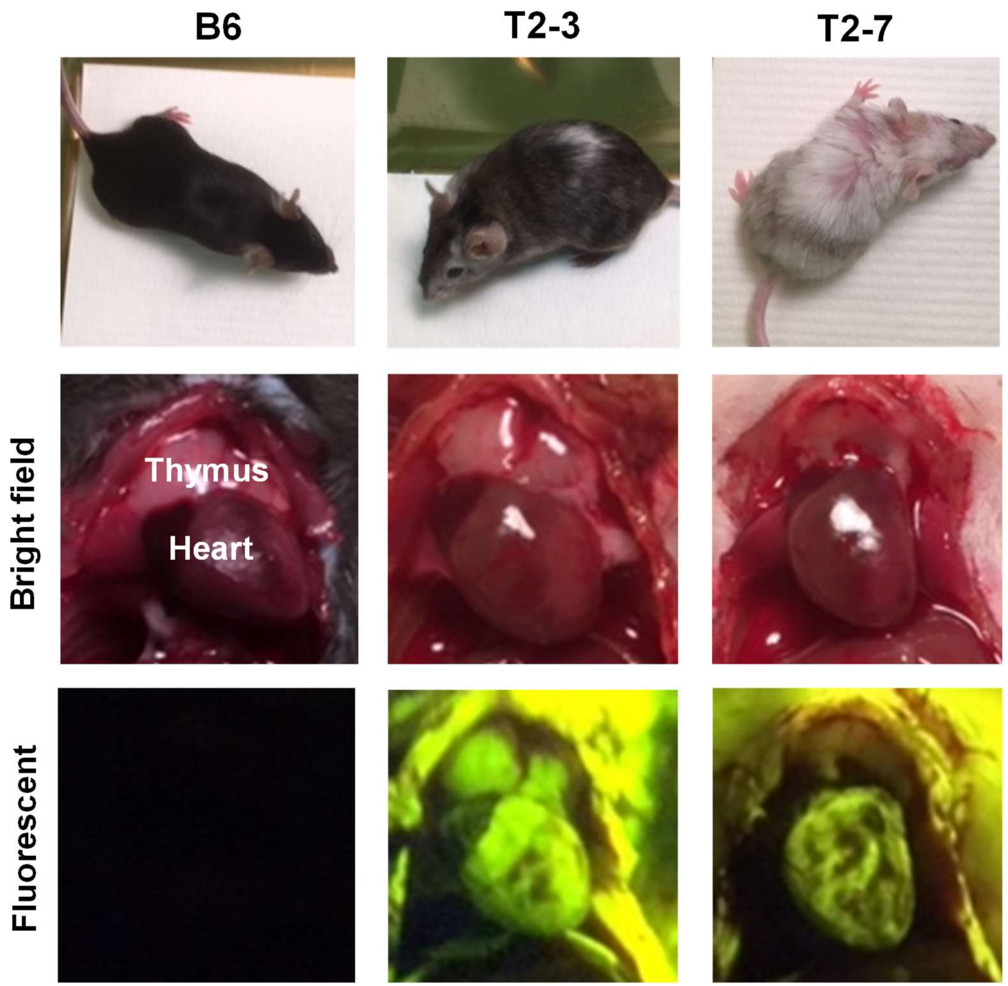

Supplementary Figure 1. Representative outward appearance and thymi of C57BL/6NCrSlc (B6) and B6 ESC$^{\text{CAG-EGFP}}$ → *Foxn1*$^{nu/nu}$ chimeric mice (mice nos. T2-3 and T2-7).

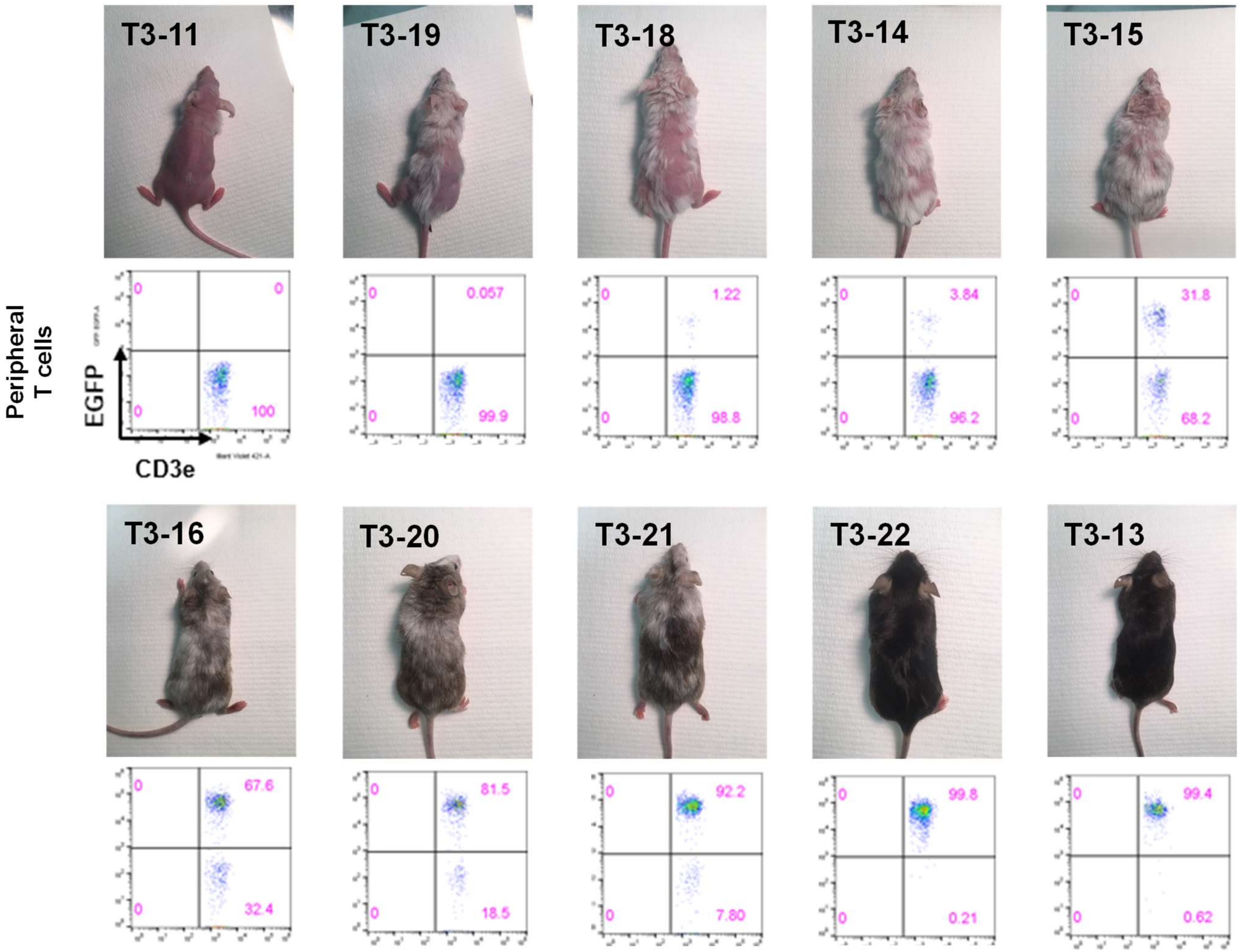

Supplementary Figure 2. Outward appearance and chimerism of peripheral T cells of 12 C57BL/6 ESC$^{CAG\text{-}EGFP}$ → *Foxn1$^{nu/nu}$* chimeric mice with varying chimerism. T cells were analyzed by flow cytometry (FCM) sorting against CD3 and EGFP intensity to show chimerism. Results of FCM are depicted as density plots, in which percentage of cells in each of four divided sections is indicated. Individual numbers of chimeras are in their images.

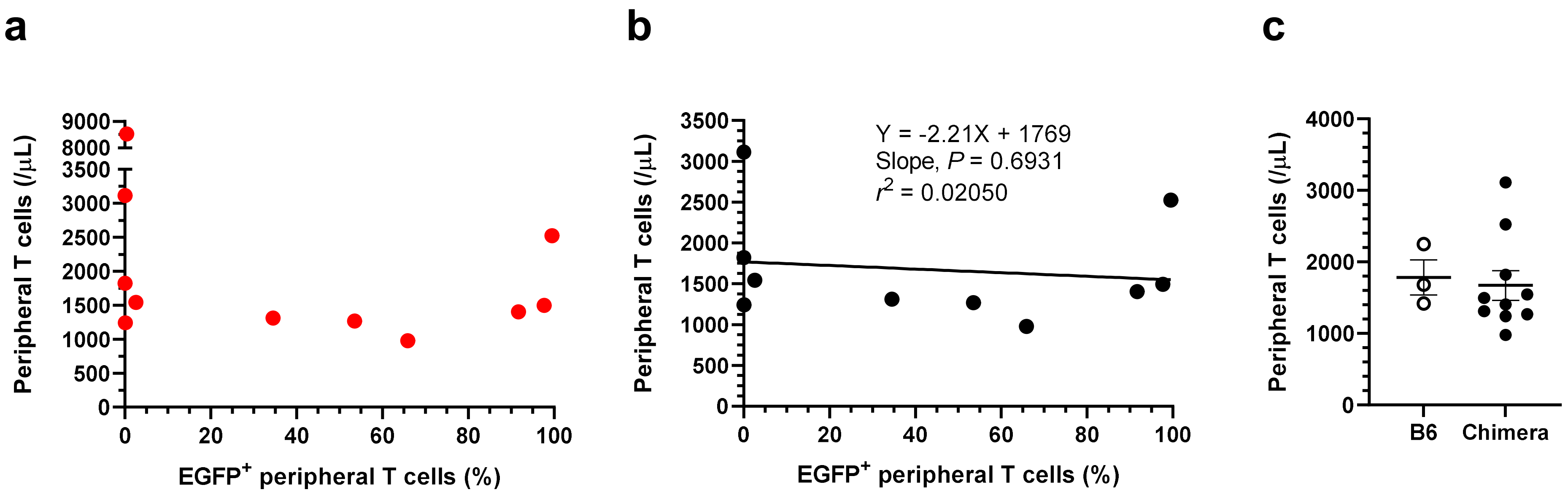

Supplementary Figure 3. Relationship between peripheral T cell numbers and chimerism of C57BL/6 ESC$^{CAG\text{-}EGFP}$ → *Foxn1*$^{nu/nu}$ chimeric mice. (**a**) Peripheral T cell numbers and percentages of EGFP^{+} peripheral T cells of 11 chimeras are plotted. One chimera shows extremely high peripheral T cell number value (~8500 /μL), which is regarded as an outlier by Grubbs's test for outliers ($P < 0.05$). (**b**) A regression line is drawn after excluding the outliner, showing no correlation between chimerism and peripheral T cell numbers. (**c**) Comparison of peripheral T cell numbers between C57BL/6JJcl (B6) and chimeric mice after excluding the outlier. Values are expressed as means ± S.E.M. There is no significant difference of peripheral T cell numbers between B6 and chimeras ($P = 0.7881$ by unpaired two-tailed Student's *t*-test). B6, n = 3; chimeras, n = 10.

Supplementary Table 1. List of antibodies used for flow cytometry studies

Antigen	Fluorochrome	Vendor	Cat No.	Isotype	Clone	Dilution rate
Mouse CD45.2	PE	BioLegend	109807	Mouse IgG2a, κ	104	×200
Mouse CD3ε	BV421	BioLegend	100336	Armenian Hamster IgG	145-2C11	×50
Mouse/human B220	BV711	BioLegend	103255	Rat IgG2a, κ	RA3-6B2	×200
Mouse CD8a	APC/Cy7	BioLegend	100714	Rat IgG2a, κ	53-6.7	×100
Mouse CD4	APC	BD Biosciences	561091	Rat IgG2a, κ	RM4-5	×100
Mouse CD326 (EpCAM)	PE/Cy7	BioLegend	118215	Rat IgG2a, κ	G8.8	×50
Mouse interferon γ	BV650	BD Biosciences	563854	Rat IgG1, κ	XMG1.2	×100
Mouse/human granzyme B	PE	BioLegend	372208	Mouse IgG1, κ	QA16A02	×100
Mouse interleukin-2	PE-CF594	BD Biosciences	562483	Rat IgG2b, κ	JES6-5H4	×100
−	BV650	BD Biosciences	563848	Rat IgG1, κ	R3-34	×100
−	PE	BioLegend	400139	Mouse IgG1, κ	MOPC-21	×100
−	PE-CF594	BD Biosciences	562308	Rat IgG2b, κ	A95-1	×100
Mouse CD16/32		BD Biosciences	553142	Rat IgG2b κ	2.4G2	×10
Mouse CD3	BUV737	BD Biosciences	612803	Rat IgG2b, κ	17A2	×200
Mouse CD45	PerCP-Cy5.5	BioLegend	103132	Rat IgG2b, κ	30-F11	×200
Mouse CD11b	BV786	BioLegend	101243	Rat IgG2b, κ	M1/70	×200
Mouse CD4	Alexa Fluor 700	BD Biosciences	557956	Rat IgG2a, κ	RM4-5	×200
Mouse CD279 (PD-1)	BV605	BioLegend	135220	Rat IgG2a, κ	29F1A12	×200
−	BV605	BioLegend	400540	Rat IgG2a, κ	RTK2758	×200
